# Evaluation of the diuretic activity of the aqueous and 80% methanol extracts of *Ajuga remota* Benth (Lamiaceae) leaves in mice

**DOI:** 10.1186/1472-6882-14-135

**Published:** 2014-04-10

**Authors:** Wubshet Hailu, Ephrem Engidawork

**Affiliations:** 1 Department of Pharmacology and Clinical Pharmacy, Addis Ababa University, P.O. Box 1176, Addis Ababa, Ethiopia

**Keywords:** Furosemide, Diuretics, Ajuga remota, Natriuresis, Kaliuresis

## Abstract

**Background:**

In the Ethiopian traditional medicine, the leaves of *Ajuga remota* B. (Local name, Armagusa) is used in the treatment of hypertension. Since this claim has not been investigated scientifically, the aim of the present study was to evaluate the diuretic potential of the aqueous and 80% methanol extracts of the leaves of *Ajuga remota* in mice after acute oral administration.

**Methods:**

Adult mice were administered orally either aqueous (250 mg/kg, AA250; 500 mg/kg, AA500 and 1000 mg/kg, AA1000) or 80% methanol (250 mg/kg, AM250; 500 mg/kg, AM500 and 750 mg/kg, AM750) extract. Urine output and electrolyte contents were then quantified up to 5 h and compared with those administered with furosemide 10 mg/kg (F10) and distilled water (CON).

**Results:**

The larger dose of 80% methanol extract produced significant diuresis (*p < 0.01*), while the aqueous extract had shown diuresis both at the middle (*p < 0.01)* and higher (p *< 0.01)* doses by the end of the fifth hour compared to CON mice. Regarding electrolyte excretion, larger doses of both extracts had increased natriuresis (*p < 0.001* for AA1000 and *p < 0.01* for AM1000), while the effect on kaliuresis were smaller when compared with the standard, suggesting the plant could possibly have a potassium-sparing effect. Phytochemical screening revealed the presence of secondary metabolites like phenolic compounds, tannins, saponins, flavonoids, terpenoids, steroids, and cardiac glycosides, which might account for the diuretic activity.

**Conclusions:**

The results indicate that the plant is endowed with significant diuretic activity at various doses, providing evidence for its folkloric use. The major components like flavonoids, tannins, terpenoids and alkaloids found in the plant might have contributed to the observed diuretic activity.

## Background

Medicinal plants have been known for millennia and are highly esteemed all over the world as a rich source of therapeutic agents for the prevention of diseases, and their health benefits are growing rapidly in recent time. The reason for this may be that some plants demonstrate effects comparable to the outcome obtained from allopathic medicines [1,2]. One of the application areas of botanicals is their diuretic effect, since there are an increasing number of plants having such effects. This is supported by a number of researches published that evaluated the degree of clinical support for the use of folklore medicines as diuretics [3-5].

*Ajuga remota* Benth (Lamiaceae) is an erect rhizomatous pubescent herb that belongs to the genus Ajuga and growing in the grasslands of Kenya and other parts of East Africa. In Ethiopia, the vernacular names for the plant include A*rmaguusaa* (Oromiffa) and it is not edible to animals, birds or insects. This is probably due to the very bitter taste of almost all its parts [6].

Several studies are conducted on many species of the genus Ajuga and their active compounds have been identified. These efforts have led to the isolation of a number of compounds, including phytoecdysteroids, neo-clerodane- diterpines and diterpinoids, triterpines specific sterols, beta-sitosterol, gamma-sitosterol, ceryl alcohol, anthocyanidin-glucosides and iridoid glycosides, quinols, withanolid, flavonoids, tannins, triglycerides and essential oils [7-9].

In East Africa, plants of the genus Ajuga have been used as a remedy for fever, toothache, dysentery, and high blood pressure. In North Africa, Ajuga plants are used to treat diabetes and hypertension, as a panacea (cure-all), specifically for gastrointestinal disorders, and as an anthelmintic [8]. In Ethiopian folk medicine, *Ajuga remota* is widely used traditionally to treat high blood pressure and stomach pain, particularly in Bahirdar Zuria, Amhara region [10]. Although ample ethnobotanical evidence exists for the use of *Ajuga remota* in the treatment of hypertension, the claim has not been substantiated scientifically. The present study had therefore attempted to present scientific data for the biological effects of the plant.

## Methods

### Collection of plant material

The leaves of *Ajuga remota* were collected from a place called Sebeta, a few kilometers West of Addis Ababa, Ethiopia in December 2010. Taxonomic identification was made by Ato Melaku Wondafrash and a voucher specimen deposited (Voucher Specimen number WH/001) at the National Herbarium, College of Natural Sciences, Addis Ababa University.

### Experimental animals

Adult male albino mice bred in the animal house of Ethiopian Health and Nutrition Research Institute with weights ranging from 25 to 30 g and 8 weeks of age were used for the experiment. The animals were housed in polypropylene cages (6–10 animals per cage) under standard environmental conditions (25 ± 1°C, 55 ± 5% humidity and 12 h/12 h light/dark cycle). The animals were allowed free access to tap water and laboratory pellet. The care and handling of animals were in accordance with internationally accepted guidelines for use of animals [11] and the procedure was approved by the School of Pharmacy Ethics Committee, Addis Ababa University (IRB/SoP/ 02/03/2010).

### Extraction of plant material

The leaves of *Ajuga remota* were sliced to smaller pieces and dried at room temperature under shade. The dried and sliced pieces of the leaves were then powdered finely and subjected to extraction.

#### Aqueous extract

Two hundred gram of the dried and powdered leaves of *Ajuga remota* was boiled at 100°C in 1000 ml of distilled water for 30 min, as used traditionally, and cooled to room temperature for 15 min. The decoction obtained was centrifuged, filtered, and placed in an oven until dried. The dried aqueous extract was collected and weighed. The approximate yield of the dry extract was 11.5% (w/w). The dried plant extract was reconstituted with distilled water for oral administration.

#### 80% methanol extract

**Figure 1:**
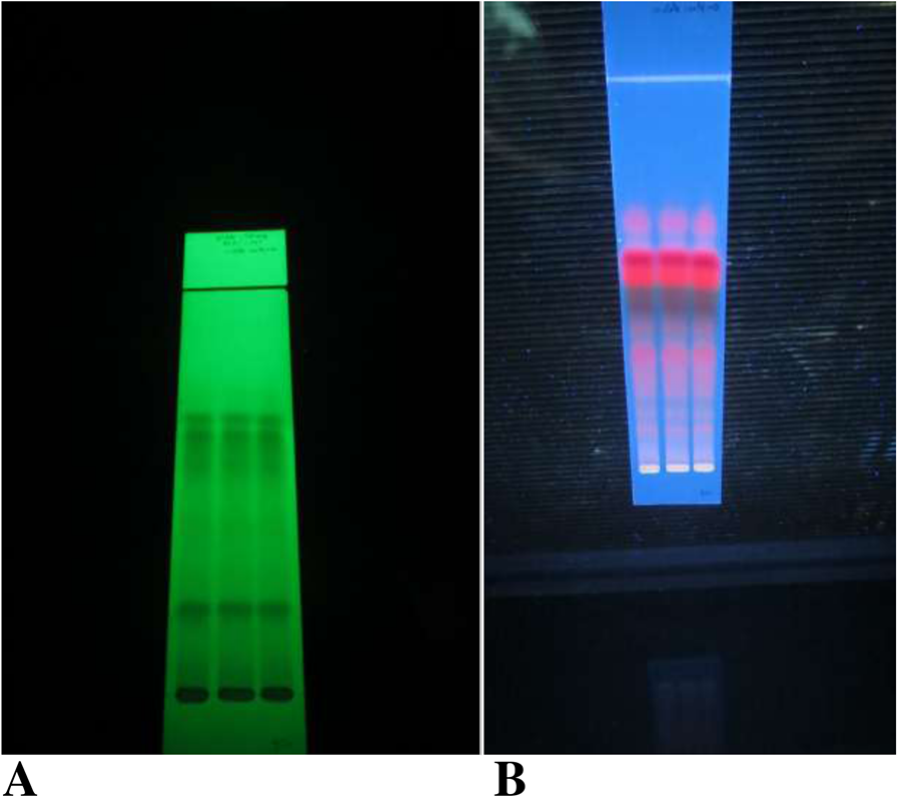
**Thin layer chromatography fingerprint of 80% methanolic leaf extract of Ajuga remota B.** The solvent system is Ethyl acetate: Toluene (85.5:14.5). **A**: when the plate is obseved under UV light of short wavelength (254 nm); **B**: when observed under UV light of long wavelength (366 nm).

Four hundred gram of the leaves of *Ajuga remota* was macerated with about 600 ml of 80% methanol for 24 h. The extract was then filtered and the marc was remacerated twice using the same volume of solvent to exhaustively extract the plant material. The methanol was then removed from the extract by evaporation under reduced pressure using a rota vapor (BUCHI Rotavapour R-200, Switzerland) at 40°C. The resulting dry hydroalcoholic extract was weighed and calculated for percentage yield, which was 9.95% (w/w). The dried plant extract was reconstituted with distilled water for oral administration. Thin layer chromatography was run to produce a fingerprint of the 80% methanol extract of the leaves of Ajuga remota and the fingerprint is depicted in Figure 1.

### Acute toxicity test

Five groups of mice formed and received orally different doses (350–5000 mg/kg) of the aqueous or hydroalcoholic extract. The animals were observed for overt toxicities like diarrhea, weight loss, tremor, lethargy and paralysis periodically for the first four hours during the 24 h period and then followed for 14 days for any lethality [12].

### Grouping and dosing of animals

Animals were randomly assigned into eight groups each consisting of 8 male mice for diuretic test. Negative controls were treated with the vehicle used for reconstitution (2 ml/100 g of body weight, CON), according to the OECD guideline. Positive controls were treated with standard drug, furosemide 10 mg/kg (F10). Three treatment groups in each test were treated with different doses of the extract as follows: the aqueous extract with doses of 250 mg/kg (AA250), 500 mg/kg (AA500), and 1000 mg/kg (AA1000); and hydroalcoholic extract with doses of 250 mg/kg (AM250), 500 mg/kg (AM500), and 750 mg/kg (AM750). Dose selection was made based on the acute toxicity test performed prior to commencement of the actual experiment. For the aqueous extract, one tenth of the limit dose was taken as the middle dose (500 mg), half of it as the lower dose (250 mg) and twice of the middle dose as the larger dose (1000 mg). For the hydroalcoholic extract, the same approach was followed for the lower and middle dose but 1.5 fold of the middle dose was set as the larger dose. This was done because the hydroalcolic extract was positive for many of the secondary metabolites.

### Diuretic activity

Diuretic activity was determined following the methods used by Lahlou et al. [13], with slight modification. Each male mouse was placed in an individual metabolic cage (Metabolic cage for mice, TECHNIPLAST, Italy) 24 h prior to commencement of the experiment for adaptation and then fasted overnight with free access to water. The animals were pretreated with physiological saline (0.9% NaCl) at an oral dose of 0.15 ml/10 g body weight, to impose a uniform water and salt load [14]. Each group was then treated as described in grouping and dosing section orally by gavage. Immediately after administration, the mice were individually placed in a metabolic cage. Urine was then collected and measured 1, 2, 3, 4, and 5 h after dosing. The urine was then filtered and finally stored at −20°C for electrolyte analyses [15].

The following parameters were calculated in order to compare the effects of the extracts with vehicle and standard on urine excretion. The urinary excretion independent of the animal weight was calculated as total urinary output divided by total liquid administered (Formula −1). The ratio of urinary excretion in test group to urinary excretion in the control group was used as a measure of diuretic action of a given dose of a drug (Formula −2). A parameter known as diuretic activity was also calculated. To obtain diuretic activity, the diuretic action of the extract was compared to that of the standard drug in the test group (Formula – 3) [14].

Formulae

(1) $$\mathrm{Urinary}\;\mathrm{Excretion}=\frac{\mathrm{Total}\;\mathrm{urinary}\;\mathrm{output}}{\mathrm{Total}\;\mathrm{liquid}\;\mathrm{administered}}\times 100\%$$

(2) $$\mathrm{Diuretic}\;\mathrm{Action}=\;\frac{\mathrm{Urinary}\;\mathrm{excretion}\;\mathrm{of}\;\mathrm{treatment}\;\mathrm{groups}}{\mathrm{Urinary}\;\mathrm{excretion}\;\mathrm{of}\;\mathrm{control}\;\mathrm{group}}$$

(3) $$\mathrm{Diuretic}\;\mathrm{Activity}=\frac{\mathrm{Diuretic}\;\mathrm{action}\;\mathrm{of}\;\mathrm{test}\;\mathrm{drug}}{\mathrm{Diuretic}\;\mathrm{action}\;\mathrm{of}\;\mathrm{standard}\;\mathrm{drug}}$$

### Analytical procedures

Sodium, potassium and chloride levels of urine and the plant extract were analyzed. Sodium and potassium concentrations were determined by making use of flame photometry, and chloride concentration was quantified using Ion Selective Electrode (ISE) analyzer (AVL 9181 Electrolyte Analyzer, Roche, USA). The flame photometer worked by flame production when the atom changed from its excited state to the ground state, while the ISE analyzer contains software which permits electrolyte parameter configuration. A calibration was performed automatically in both cases prior to analysis with different levels of standards. Ratios of electrolytes; Na^+^/K^+^ and Cl^−^/K^+^+Na^+^ were calculated to evaluate the saluretic activity of the different extracts. In addition, pH was directly determined on fresh urine samples using a pH-meter. Moreover, the salt content of the extract was also determined to rule out its contribution on urinary electrolyte concentration.

### Phytochemical screening

Phytochemical screening tests were carried out for the aqueous and 80% methanol extracts of the plant using standard procedures [16] to identify the presence of secondary metabolites, including phenolic compounds, tannins, saponins, flavonoids, terpenoids, steroids, alkaloids, anthraquinones and cardiac glycosides.

### Statistical analysis

Data are expressed as mean ± S.E.M (standard error of mean). Statistical analysis of the data were performed with one-way analysis of variance (ANOVA) followed by Tukey’s multiple comparison test. Significant differences were set at *P* values lower than 0.05.

## Results

### Acute toxicity study

Mice were observed for two weeks to see if the aqueous and hydroalcoholic plant extracts had an acute toxic effect. Both extracts of the plant did not produce any visible signs of toxicity up to the dose of 5 g/kg. This was evidenced by absence of tremor, loss of weight, lethargy, paralysis, stress or adverse behaviors. In addition, there was also no sign of diarrhea and none of the treated mice died, suggesting the LD50 is greater than 5000 mg/kg.

### Diuretic activity: effect on urine volume

#### Aqueous extract

**Figure 2:**
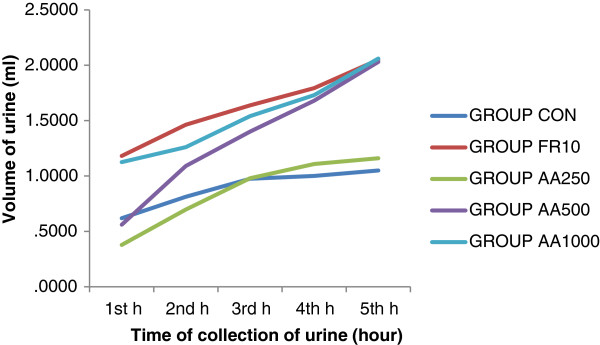
**Time course of diuresis in mice treated with different doses of aqueous extract of the leaves of *Ajuga remota*: n = 8, AA250 - 250 mg/kg, AA500 - 500 mg/kg, and AA1000 - 1000 mg/kg of aqueous extract of *Ajuga remota*; F10- Furosemide 10 mg/kg, CON – group that received distilled water.**

The aqueous extract of the plant produced diuresis, which appeared to be a function of dose (Table 1). The time course of action of diuresis is also depicted in Figure 2. AA250 did not produce any detectable difference in urine volume compared to CON animals at all time points. AA500 started to increase urine volume from the second hour, but changes reached statistical significance at the fourth (68%, p < 0.05) and fifth (93.3%, p < 0.01) hour. By contrast, the increase in urine volume with the highest dose (AA1000) was significant (82.2%, p < 0.01) starting from the very first hour and a maximum increase was noted at the fifth hour (96%, p < 0.01). F10 treated mice exhibited a significantly greater urine volume from the first hour (90%, p < 0.01), which continued until the end of the fifth hour (95%, p < 0.01). F10 displayed a significantly greater effect compared to AA250 (p < 0.001) throughout the time points, but had no significant difference with that of AA500 from the second hour onwards. Comparable effects were observed between F10 and AA1000 across all time points.

**Table 1:** **Effect of aqueous and 80% methanol extracts of the leaves of *Ajuga remota *** **on 5 h urine volume in mice**

**Group**	**Volume of urine (ml)**					**Diuretic action**	**Diuretic activity**
	**1 h**	**2 h**	**3 h**	**4 h**	**5 h**		
CON	0.62 ± 0.13	0.81 ± 0.11	0.97 ± 0.11	1.0 ± 0.11	1.05 ± 0.10	1.0	
F10	1.18 ± 0.07^a1^	1.46 ± 0.09^a3^	1.64 ± 0.08^a1^	1.79 ± 0.09^a2^	2.05 ± 0.11^a2^	1.95	1.0
AA250	0.38 ± 0.04^b3,c3^	0.69 ± 0.05^b3,c2^	0.98 ± 0.17^b2^	1.11 ± 0.18^b2,c1^	1.16 ± 0.17^b3,c2,d1^	1.10	0.56
AA500	0.56 ± 0.08^b3,c2^	1.09 ± 0.07	1.41 ± 0.12	1.68 ± 0.13^a1^	2.03 ± 0.13^a2^	1.93	0.99
AA1000	1.13 ± 0.13^a2,g3,h3^	1.26 ± 0.14^a1,f3,g3^	1.54 ± 0.19^a1^	1.73 ± 0.17^a2^	2.06 ± 0.20^a2^	1.96	1.01
AM250	0.45 ± 0.46^b3,c2^	0.62 ± 0.07^b3^	0.68 ± 0.72^b3,c2^	0.84 ± 0.07^b3,c2^	1.1 ± 0.85^b3,c3^	1.06	0.54
AM500	0.48 ± 0.09^b3,c1^	0.71 ± 0.09^b3^	0.91 ± 0.12^b3^	1.08 ± 0.13^b2,c1^	1.31 ± 0.11^b2,c2^	1.25	0.64
AM750	0.86 ± 0.03^e3,d3^	0.98 ± 0.08^e3^	1.32 ± 0.13	1.57 ± 0.16^a1^	2.01 ± 0.19^a2^	1.92	0.98

(n = 8); For aqueous extract: ^a^against control, ^b^against standard, ^c^against AA1000 mg/kg, ^d^against AA500 mg/kg, ^e^against AA250, ^f^against AM250, ^g^against AM500, ^h^against AM750; For 80% methanol extract ^a^against control, ^b^against standard, ^c^against AM750 mg/kg; ^1^p < 0.05, ^2^p < 0.01, ^3^p < 0.001; AA refers to aqueous extract and AM to 80% methanol extract Ajuga remota; F10: Furosemide 10 mg/kg CON: controls treated with distilled water; Numbers refer dose in mg/kg; Diuretic activity is considered to be good if it is more than 1.50, moderate if it is 1.00-1.50, little if it is between 0.72-1.00 and nil if it less than 0.72.

#### Hydroalcoholic extract

No apparent difference was observed between controls and mice treated with the first two doses (AM250 and AM500) at all time points. The larger dose (AM750) although resulted in an increased urine volume compared to CON mice starting from the first hour (38.7%), it failed to reach statistical significance. However, the change became significant at the fourth hour (57%, p < 0.05), with a maximum effect at the fifth hour (91.4%, p < 0.01) when compared with the CON mice (Table 1). F10, on the other hand, produced a significant increase starting from the first hour.

Both AM250 (p < 0.001) and AM500 (p < 0.01) produced a lower diuretic effect as compared to that of F10, while the maximum dose (AM750) had an effect which was comparable to that of F10 (Table 1).

### Saluretic activity: effect on electrolyte content of the urine

#### Aqueous extract

The urine samples collected over the five hours were analyzed for the electrolyte content (Na^+^, K^+^, and Cl^−^) and presented in Table 2. Whilst AA250 tended to decrease sodium loss by 17.1%, AA500 tended to increase by 34.4% compared to CON group. By contrast, AA1000 and F10 significantly increased sodium loss by 69.2% (p < 0.001) and 68.9% (p < 0.001), respectively, compared to CON animals.

**Table 2:** **Effect of aqueous and 80% methanol extracts of the leaves of *Ajuga remota *** **on 5 h urinary electrolyte excretion in mice**

**Group**	**Urinary electrolyte concentration (mmol/L)**			**Saluretic index**			**Na**^**+**^**/K**^**+**^	**Cl**^**−**^**/ Na**^**+**^**+K**^**+**^
	**Na**^**+**^	**K**^**+**^	**Cl**^**−**^	**Na**^**+**^	**K**^**+**^	**Cl**^**−**^		
DW	59.97 ± 7.83	43.7 ± 5.80	44.2 ± 2.58				1.37	0.43
F10	101.3 ± 6.01^a3^	92.07 ± 13.85^a3^	73.85 ± 11.27	1.68	2.1	1.67	1.10	0.38
AA250	49.70 ± 2.74^b3,c3^	57.5 ± 5.94^b1^	50.87 ± 10.58	0.83	1.32	1.13	0.86	0.47
AA500	80.62 ± 4.89	72.85 ± 2.66^c1^	58.75 ± 8.34	1.34	1.67	1.33	1.11	0.38
AA1000	101.5 ± 6.98^a3^	38.08 ± 1.81^b3^	83.37 ± 2.71^a3^	1.69	0.87	1.87	2.66	0.59
AM250	63.75 ± 7.3^b1,d1^	97.5 ± 13.2^c1^	55.74 ± 6.23	1.06	2.23	1.26	0.65	0.34
AM500	90.37 ± 5.8^a1,e1^	100.6 ± 11.7^c2^	56.82 ± 8.06	1.51	2.30	1.29	0.89	0.29
AM750	101.7 ± 4.72^a2,e2^	53.5 ± 2.95	77.78 ± 9.71^a1^	1.69	1.22	1.76	1.90	0.50

(n = 8); For aqueous extract ^a^against control, ^b^against standard, ^c^against AA1000 mg/kg; For 80% methanol extract, ^a^against control, ^b^against standard, ^c^against AM750 mg/kg, ^d^against AM500 mg/kg, ^e^against AM250 mg/kg; ^1^p < 0.05, ^2^p < 0.01, ^3^p < 0.001; Saluretic Index = mmol of electrolyte of test group/mmol of electrolyte of control group; AA refers to aqueous extract and AM to 80% methanol extract of Ajuga remota; F10: Furosemide 10 mg/kg CON: controls treated with distilled water; Numbers refer dose in mg/kg.

Urinary K^+^ excretion was significantly higher with F10 (106.8%, p < 0.001) compared to CON group. AA250 and AA500 tended to increase kaliuresis by 31.6% and 66.7%, respectively, which was not statistically significant compared to CON mice. The larger dose of the aqueous extract, on the other hand, reduced K^+^ loss by 8.8% compared to controls, but the decrease was significant (41.4%, p < 0.001) when compared to F10. In the case of Cl^−^ excretion, though treatment tended to increase loss of the anion compared to CON mice, the increase reached statistical significance only with AA1000 (88.6%, p < 0.001).

#### Hydroalcoholic extract

The urinary Na^+^ excretion inclined to exhibit an increasing pattern with dose, as it was 6.3%, 50.7% and 69.6% (p < 0.01) for AM250, AM500 and AM750, respectively, when compared to CON group (Table 2). On the other hand, K^+^ excretion had insignificantly increased from AM250 to AM500 but came down below the control level, albeit insignificantly, with AM750. The excretion profile of Cl^−^ had also increased by 26.1%, 28.6%, and 76% (p < 0.05) for AM250, AM500 and AM750, respectively (Table 2).

Na^+^ loss produced by F10 was significantly greater compared to CON (p < 0.001) and AM250 (p < 0.05), but no apparent difference was noted when compared to AM500 and AM750 groups. K^+^ excretion, however, appeared to be insignificantly higher with the lower and middle dose of the extract, but lower with the highest dose used in this study compared to F10 mice. In addition, no apparent difference was observed in Cl^−^ excretion between the standard and all doses of the extract.

### Electrolyte content of the extract

Water soluble salts could be present in the extract and subsequently interfere with the urinary excretion of electrolytes. The content of Na^+^, K^+^ and Cl^−^ in both extracts was therefore determined to exclude this possibility. The result revealed that Na^+^ and Cl^−^ were not detectable at all doses in the extracts as tested by the instrument used in the present study. K + content of the aqueous extract was found to be 35.3, 40.7 and 47.1 mmol/l for AA250, AA500 and AA1000, respectively. In case of 80% methanol extract, values as low as 12, 20 and 23.5 mmol/l was detected for AM250, AM500 and AM750, respectively.

### Urinary pH

**Figure 3:**
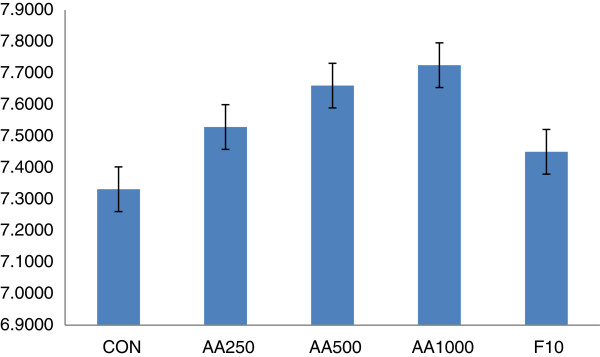
**Urinary pH of mice treated with aqueous extract of the leaves of *Ajuga remota*: AA250- 250 mg/kg, AA500- 500 mg/kg, and AA1000- 1000 mg/kg of aqueous extract of *Ajuga remota*; F10- Furosemide 10 mg/kg, CON –group that received distilled water.**

**Figure 4:**
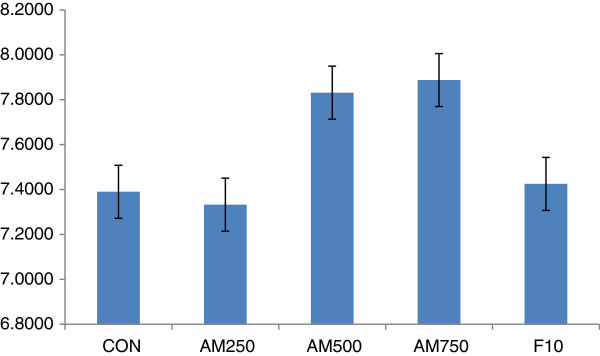
**Urinary pH of mice treated with 80% methanol extract of the leaves *Ajuga remota*: AM 250–250 mg/kg, AM 500–500 mg/kg, and AM 1000–1000 mg/kg of 80% methanol extract of *Ajuga remota*; F10- Furosemide 10 mg/kg, CON –group that received distilled water.**

Urinary pH measurement revealed that the different treatment groups of both aqueous and 80% methanol extracts had produced relatively alkaline urine. The pH of urine treated with the aqueous extract had shown an increase from AA250 (7.57) to AA1000 (7.72). The CON group produced the lowest pH and the standard group an intermediate pH (7.45) between vehicle and extract treated groups (Figure 3). Treatment with the hydroalcoholic extract increased pH from 7.3 (AM250) to 7.88 (AM750). Urinary pH of CON and F10 was higher than the lowest doses but lower than the other doses (Figure 4).

### Phytochemical screening

Both extracts of *Ajuga remota* were explored for the composition of medicinally active compounds and both were found to be positive for phenolic compounds, saponins, steroids, cardiac glycosides, flavonoids, tannins and terpenoids. On the other hand, alkaloids and anthraquinones were only found in the hydroalcoholic extract.

## Discussion

Diuretic activity may be very useful in a number of conditions like hypertension, hypercalciuria, and cirrhosis of liver. Since diuretics are employed clinically in the treatment of edema, it would be highly important to demonstrate effectiveness in the presence of electrolyte and water [17]. Thus, it is presumed to be advantageous to ‘pre-treat’ or ‘prime’ the test animals with various fluids in screening agents for potential diuretic activity.

The aqueous and 80% methanol extracts of the leaves of *Ajuga remota* showed an increase in urine volume that appeared to vary with dose and time as well as the nature of the extract. Compared to the 80% mehanol extract, the aqueous extract produced a better diuretic effect, particularly with increasing dose in the early hours (p < 0.05 for AA1000 vs. AM250, AM500, & AM7500) (Table 1). The lower doses of both extracts did not produce an appreciable effect, but, whilst the medium dose of the aqueous extract was able to produce significant effect beginning from the fourth hour, the same dose of the 80% extract was devoid of any effect until the end of the observation period. This could probably suggest that the lower doses might represent subthreshold doses. The higher dose of both the aqueous and 80% methanol extracts produced comparable effect to that of furosemide. Even better effect was observed with the aqueous extract, as it had a diuretic activity even more than the standard drug. It is therefore possible to suggest that the ingredient (s) of the plant material responsible for increasing urine output could probably be polar and hence better extracted in water than 80% methanol.

The diuretic activity of the extracts of *Ajuga remota* at their higher respective doses was a moderate type for the aqueous and mild for the hydroalcoholic extract, since their values were 1.01 and 0.98 for AA1000 and AM750, respectively. Diuretic activity is considered to be good if it is more than 1.50, moderate if it is 1.00-1.50, little if it is between 0.72-1.00 and nil if it less than 0.72 [18].

The effect of the extracts on water excretion was accompanied by urinary electrolyte excretion effect, since there appeared to be an increased salt excretion as compared to the control group, which supports the idea that the diuretic effect of *Ajuga remota* was of the saluretic type in contrast to aquaretic type, which is a typical feature of most phytodiuretic agents [19]. The larger doses of both extracts did have an interesting natriuretic effect and thus could have a beneficial effect in different edematous conditions. The ratio Na^+^/K^+^ was calculated as indicator of natriuretic activity and resulted in values of 2.66, 1.90 and 1.1 for AA1000, AM750, and F10 respectively. This indicates that the extracts increase sodium excretion more than potassium, which is considered as a very good safety profile of diuretic agents, as hypokalemia is one of the potential adverse effects of synthetic diuretics, such as furosemide.

In contrast to previous studies of aqueous and methanol extracts of some plants which showed an interesting K^+^-saving effect at low and intermediate doses [19], in the present study this effect was observed at the higher doses. It is probable that at low dosages of the aqueous and methanol extracts of *Ajuga remota*, the substances responsible for K^+^- sparing effect might not reach sufficient concentration to exert the effect. On the other hand, aqueous and 80% methanol extracts of some plants such as *Rumex abyssinicus* J. have been demonstrated to be devoid of K^+^- sparing effect [5]. These observations point to the fact that there are at least two different mechanisms by which diuersis could be achieved, one of which produces notable diuresis with a sparing of potassium and another with very strong diuresis in which there is a clear tendency to lose the K^+^ - conservative effect [20]*.*

Moreover, onset of the diuretic action of AA1000 was sufficiently rapid and had a fairly long duration of action as it produced significant effect from the first hour (p < 0.01) to the fifth hour (p < 0.01) of the experiment. This is an appealing diuretic profile, as it would curtail the frequency of administration. This coupled to the little or no risk of hypokalemia makes the extract a potential herb worth investigating for treatment of edematous conditions.

It is possible that *Ajuga remota* extracts exerted diuretic effect by inhibiting tubular reabsorption of water and electrolytes, since such action has been suggested for some other plants. The possibility of direct action of potassium content of the plant extract on diuretic effect could be excluded, since the K^+^ content of the extract was very low in comparison with the salt concentration obtained from other plants [21].

Loop diuretics like furosemide increase urinary flow rate and urinary excretion of sodium, potassium and chloride by inhibiting Na^+^–K^+^–2Cl^−^ symporter in the thick ascending loop and by inhibiting carbonic anhydrase enzyme*.* The larger doses of aqueous and 80% methanol extracts of *Ajuga remota* used in the present study produced similar Na^+^ and Cl^−^ excretion profile to that of furosemide. However, there is a difference between the extracts and the standard when K^+^ excretion is considered. This could possibly suggest that the mechanism by which the extract produces diuresis is not exactly the same to that of loop diuretics. It is also possible to exclude the thiazide-like type of mechanism either, as the extracts, depending on dose, relatively increased the urinary K^+^ level more and alter the urinary Na^+^/K^+^ ratio.

The Cl^−^/ Na^+^+K^+^ ratio was calculated and shows the extent of carbonic anhydrase inhibitory effect. Carbonic anhydrase inhibition can be excluded at ratios between 0.8 and 1.0 and with decreasing ratios; slight to strong inhibition can be assumed [11]. The Cl^−^/ Na^+^+K^+^ ratio was calculated for both extracts and the intermediate doses, AA500 and AM500, showed the strongest inhibitory effect with values of 0.38 and 0.29, respectively. Thus, it appears likely that the strongest carbonic anhydrase inhibition effect at these middle doses might have contributed to the highest K^+^ loss compared to the other doses. Hence, it is plausible to assume that one of the possible mechanisms of action of these extracts could be carbonic anhydrase inhibition.

In determination of urinary pH, the extracts showed a relative increase in pH values as compared to controls, reinforcing the notion that carbonic anhydrase inhibition as one of the possible mechanism of action of the plant. In addition, the reduction of potassium excretion at the maximum doses of the extracts along with the resulted alkalinization of urine might give a clue on the possibility of the plant acting as a modest potassium-saving diuretic.

The exact nature of the active principle/s responsible for the diuretic effects of the hydroalcoholic and aqueous extracts of the plant is/are, so far, not known. However, preliminary phytochemical analysis carried out with both extracts revealed the presence of bioactive molecules, including flavonoids and steroids. Previous studies have demonstrated that there are several compounds which could be responsible for the plants diuretic effects such as flavonoids, saponins or organic acids [22]. The effect may be produced by stimulating regional blood flow or initial vasodilatation, or by producing inhibition of tubular reabsorption of water and anions, with the result in both cases being diuresis [20].

## Conclusions

Collectively, these observations provide evidence for the ethnomedical use of *Ajuga remota* for treatment of hypertension largely through enhancement of salt and water excretion. Although the active components (s) for the observed effect remain (s) to be seen, polar constituents singly or in synergy act by multiple mechanisms to produce the observed effect. Indeed, multiple mode of action had been reported with some herbal medications [23].

Looking at the data, the larger doses, particularly of the aqueous extract produced a very interesting profile, which was comparable to the loop diuretics. The safety profile of the extract is an added advantage that calls for conducting further research to ascertain the findings reported in this study.

## Competing interests

The authors declare that they have no competing interest to disclose.

## Authors’ contributions

All authors involved in the design and write up of the study, and WH conducted the actual study and the statistical analysis. Both authors approved the submitted version of the manuscript.

## Pre-publication history

The pre-publication history for this paper can be accessed here:

http://www.biomedcentral.com/1472-6882/14/135/prepub
